# Epithelial plasticity in cancer: beyond metastasis

**DOI:** 10.18632/aging.101367

**Published:** 2018-01-22

**Authors:** Timothy M. Thomson, Pedro L. Fernández

**Affiliations:** 1 Institute for Molecular Biology of Barcelona, National Research Council, Barcelona, Spain; 2 Networked Biomedical Research Center for Hepatic and Digestive Diseases (CIBER-EHD), Madrid, Spain; 3 Department of Pathology, Hospital Clínic de Barcelona, August Pi i Sunyer Biomedical Research Institute (IDIBAPS) and Universitat de Barcelona, Barcelona, Spain

**Keywords:** cancer, epithelial-mesenchymal plasticity, lineage plasticity

Recent evidence has challenged the notion that multipotent stem cell progeny follow irreversible differentiation paths along committed lineages. Thus, it has been demonstrated that dedicated adult stem cells that reside in a given tissue are not irreplaceable, given that more differentiated progeny cells can revert to perform facultative stem cell functions in response to ablation of professional stem cells. Particularly in response to damage, cells committed to a given lineage can adopt differentiated profiles distinct from those corresponding to their lineages of origin, in fate conversion (*transdifferentiation* or *metaplasia*) processes that require prior *dedifferentiation* into multipotent stem cells [1].

Further to roles in development and adult tissue homeostasis, lineage plasticity underlies cancer phenotypic heterogeneity, significantly impinging upon the clinical behavior of tumors including therapeutic options and responses [2]. Notably, “bulk” cancer cells have been proposed to acquire cancer stem cell (CSC) properties upon switching to a mesenchymal gene program [3]. Subsequent refinements of this proposal indicate that only cancer cells endowed with the ability to reversibly switch between epithelial and mesenchymal gene programs display CSC properties [2]. Cells locked in strong and stable mesenchymal programs lack the necessary epigenetic plasticity to display CSC phenotypes [4].

The demonstration of cancer cell heterogeneity associated with epithelial-mesenchymal plasticity evokes further questions on phenotypic heterogeneity and the cells of origin of cancers. Beyond their abilities to invade and/or establish tumors, CSCs are multipotent and, as such, can yield progeny that, given appropriate environmental cues, at least partially differentiate along *permissive* lineages. Different oncogenic insults affecting multipotent stem cells at different stages of commitment to different lineages may drive lineage plasticity and phenotypic heterogeneity of breast and other cancers. For instance, introduction of constitutively active PIK3CA mutants in the breast epithelial basal lineage in murine models preferentially yields luminal tumors while, when introduced in the luminal lineage, it produces luminal A, luminal B, HER2-enriched or basal-like phenotypes, concomitant with evidences of metaplasia [5]. As corollaries, untimely or abnormally sustained signaling can drive lineage fate conversion, and the putative cell lineage of origin of a given cancer should not be inferred based solely on observed phenotypes.

Conventionally, epithelial-mesenchymal plasticity is not considered an integral component of lineage plasticity in epithelial tissues. As such, physiological expression of epithelial or mesenchymal gene programs at different stages of differentiation along a given epithelial lineage would reflect gene program subsets inherent to a particular stage and lineage. Exogenous forcing of a mesenchymal gene program, e.g. in response to environmental cues, would transiently derail the lineage differentiation process, which would resume at the same point in differentiation once the mesenchymal forcing subsides. Disputing this perspective, it was recently shown that induction of an epithelial gene program through microRNA-200 (miR-200) in otherwise non-committed mammary epithelial cells drives a switch to the luminal lineage [6,7], while expression of the mammary basal epithelial transcription factor Δp63 induces a myoepithelial differentiation [6], suggesting that epithelial or mesenchymal gene programs directly modulate lineage commitment and plasticity. Interestingly, miR-200 also boosts self-renewal properties via activation of the PI3K-AKT-mTOR signaling axis and drives the growth of tumors in xenograft models that display lineage heterogeneity, including double-lineage-positive (luminal and basal) cells [7]. Of note, miR-200 might have a driver role in the acquisition of the metaplastic phenotype in some breast carcinomas and could also serve as a blood-borne tumor progression marker in a subtype of breast neoplasms [7].

It might be timely to reconsider epithelial-mesenchymal plasticity as a significant actor in epithelial cell lineage plasticity The implications for cancer phenotypic heterogeneity, beyond “mere” epithelial-mesenchymal transition, are relevant: tumor phenotypes, including those considered “intrinsic”, can be interpreted as the combined result of the cell lineage of origin, the type, strength and timing of oncogenic insults and the engagement of epithelial or mesenchymal gene programs as readouts of environmental cues.

## References

[1] Blanpain C, Fuchs E. Science. 2014; 344:1242281. 10.1126/science.1242281 24926024 PMC4523269

[2] Chaffer CL, et al. Proc Natl Acad Sci USA. 2011; 108:7950–55. 10.1073/pnas.1102454108 21498687 PMC3093533

[3] Mani SA, et al. Cell. 2008; 133:704–15. 10.1016/j.cell.2008.03.027 18485877 PMC2728032

[4] Celià-Terrassa T, et al. J Clin Invest. 2012; 122:1849–68. 10.1172/JCI59218 22505459 PMC3366719

[5] Van Keymeulen A, et al. Nature. 2015; 525:119–23. 10.1038/nature14665 26266985

[6] Hilmarsdóttir B, et al. Dev Biol. 2015; 403:150–61. 10.1016/j.ydbio.2015.05.007 25967125

[7] Sánchez-Cid L, et al. Oncotarget. 2017; 8:83384–406. 10.18632/oncotarget.20698 29137351 PMC5663523

